# A Multimodal Approach to Diagnosis of Immunobullous Diseases: Integrating Clinical, Histopathological, and Immunofluorescence Findings

**DOI:** 10.7759/cureus.84634

**Published:** 2025-05-22

**Authors:** Abhishek De, Disha Chakraborty, Namratha Grisilda, Subhra Dhar

**Affiliations:** 1 Dermatology, Calcutta National Medical College and Hospital, Kolkata, IND; 2 Dermatology and Rheumatology, Sacramento VA Medical Center, Sacramento, USA; 3 Epidemiology, Indian Institute of Public Health, Hyderabad, IND; 4 Pathology, Wizderm Dermatopathology Lab, Kolkata, IND

**Keywords:** autoimmune bullous dermatoses, bullous pemphigoid, direct immunofluorescence, immunobullous diseases, pemphigus vulgaris

## Abstract

Introduction: Autoimmune bullous dermatoses (ABD) are rare but potentially debilitating skin disorders characterized by autoantibody-mediated blistering of the skin and mucosa. Timely and accurate diagnosis is critical for effective treatment and improving patient outcomes. To evaluate the diagnostic concordance between clinical assessment, histopathology (HP), and direct immunofluorescence (DIF) in patients with ABD and to examine the demographic and clinical patterns in a tertiary dermatology center in eastern India.

Methods: This retrospective, cross-sectional study analyzed 196 cases of ABD diagnosed between January 2020 and August 2023. Clinical findings were correlated with histopathological examination (HPE) and DIF patterns to establish final diagnoses. Concordance between different diagnostic modalities was statistically assessed using McNemar’s test.

Results: Bullous pemphigoid (BP) was the most prevalent diagnosis (58.1%), followed by pemphigus vulgaris (PV; 20.9%) and pemphigus foliaceus (PF; 11.2%). In the geriatric population, BP was predominant (81.1%), while PV and PF were more common in females. The highest diagnostic concordance (97.9%) was observed when clinical findings were combined with both HP and DIF. Four cases of epidermolysis bullosa acquisita (EBA) were diagnosed only after salt-split DIF.

Conclusion: An integrated diagnostic approach combining clinical, histopathological, and DIF findings significantly enhances diagnostic accuracy in autoimmune bullous diseases. Given the rising prevalence of BP, especially among the elderly, and the limitations of individual modalities, multimodal assessment should be the standard for optimal diagnosis and management.

## Introduction

Autoimmune bullous dermatoses (ABD) are a diverse group of rare blistering conditions marked by autoimmune responses targeting the skin and mucosal structural components, leading to blister formation. These disorders are classified into intraepidermal and subepidermal subtypes, depending on the layer of the skin where blistering occurs. Subepidermal types include bullous pemphigoid (BP), mucous membrane pemphigoid, and others, while intraepidermal forms encompass mainly pemphigus vulgaris (PV) and pemphigus foliaceus (PF) [1]. Despite their rarity, ABDs can cause significant morbidity and, in severe cases, may be life-threatening, underscoring the critical need for swift and accurate diagnosis and treatment.

Histopathology (HP) and direct immunofluorescence (DIF) are invaluable diagnostic tools in confirming ABDs. PV, the most common of these diseases, affects approximately 0.1-0.5 per 100,000 individuals globally, with PF being the second most frequent [2]. The Tzanck smear, a diagnostic method introduced in 1947 by Arnault Tzanck, remains a minimally invasive option for identifying pemphigus. However, histopathological examination (HPE) provides essential details, and DIF testing proves to be more precise, detecting bound antibodies, complement, and fibrinogen in the affected skin. [3]. Remarkably, a positive DIF test can sometimes indicate a relapse in patients who are in remission. Given the limited research in eastern India, this study seeks to explore the clinicopathological and immunofluorescence correlations in autoimmune blistering diseases, contributing valuable data to this underrepresented region.

## Materials and methods

This was a retrospective, cross-sectional observational study conducted at the dermatology department of a tertiary care teaching hospital in Kolkata, India. The study period spanned from January 2020 to August 2023.

The primary objective of this study was to evaluate the diagnostic concordance among clinical examination, histopathological analysis using hematoxylin and eosin (H&E) staining, and DIF in patients with ABD. Additionally, the study aimed to describe the demographic and clinical characteristics of the affected population and to assess the individual and combined contributions of each diagnostic modality in establishing the final diagnosis. By highlighting the diagnostic value of a multimodal approach, the study seeks to emphasize the importance of integrating clinical, histological, and immunopathological data to enhance diagnostic accuracy in routine dermatological practice.

All patients who were clinically suspected of having ABD and who underwent histopathological and/or DIF evaluation during the study period were considered for inclusion. A total of 230 patients were initially screened, of whom 196 fulfilled the eligibility criteria and were included in the final analysis.

The inclusion criteria comprised patients of any age and gender who presented with clinical features suggestive of ABD such as flaccid or tense bullae, erosions, or mucosal involvement. Eligible cases were required to have adequate skin biopsy specimens for both histopathological and DIF studies, along with complete clinical documentation including history, physical examination findings, and relevant photographic records where available.

Patients were excluded if their biopsy samples were inadequate, specifically, if they lacked intact epidermis or the dermoepidermal junction, or if prior treatment with systemic corticosteroids or immunosuppressive agents within seven days of biopsy had potentially compromised the results. Cases with missing or incomplete clinical data or documentation were also excluded. Furthermore, individuals diagnosed with non-autoimmune bullous diseases or infectious conditions mimicking bullous disorders were not considered for inclusion.

Since this was a retrospective study, a formal sample size calculation was not undertaken. Instead, a convenience sampling method was used, whereby all eligible patients during the designated study period were included. The sample size of 196 was considered adequate to conduct meaningful statistical comparisons and subgroup analyses.

The parameters extracted from clinical records and biopsy reports included demographic characteristics such as age and gender; clinical characteristics including the type and distribution of lesions, mucosal involvement, and duration of disease; and histopathological features such as the level of blister formation, presence of acantholysis, and the predominant type of inflammatory infiltrate. DIF findings were recorded based on the location and pattern of immune deposits (intercellular, linear or granular basement membrane zone (BMZ), shaggy BMZ, or vascular), along with the intensity and type of immunoglobulin or complement deposition. Final diagnoses were made by integrating clinical findings with histopathological and DIF results.

For the purpose of subgroup analysis, patients were categorized into pediatric (under 18 years), adult (18 to 59 years), and geriatric (60 years and above) age groups.

All data were compiled using Microsoft Excel and analyzed using Stata software (version X.X). Descriptive statistics were reported as means and standard deviations for continuous variables and as frequencies and percentages for categorical variables. McNemar’s test was applied to evaluate diagnostic agreement between clinical diagnosis, histopathological interpretation, and DIF findings. This test was particularly appropriate as it is designed to analyze paired nominal data and detect differences in matched proportions. A p-value of less than 0.05 was considered statistically significant. Diagnostic concordance was calculated for various combinations of modalities, including clinical versus histopathological diagnosis, histopathological versus DIF diagnosis, and combinations of all three.

## Results

Among the initial 230 individuals screened for immunobullous diseases, 196 fulfilled the inclusion criteria. PV was the most prevalent condition in the intraepidermal group, while BP was the most common both in the subepidermal group and overall (Table 1).

**Table 1:** The percentage distribution of intraepidermal and subepidermal bullous disorders PF: Pemphigus foliaceus; PV: Pemphigus vulgaris; IgA: Immunoglobulin A; BP: Bullous pemphigoid; CBDC: Chronic bullous disease of childhood; EBA: Epidermolysis bullosa acquisita; DH: Dermatitis herpetiformis; LPP: Lichen planus pemphigoid; LIGA: Linear IgA disease

Group	Diseases	No. of cases (n%) (N=196)
Intraepidermal group (n=67)	PF	22 (11.2%)
	Pemphigus vegetans	3 (1.5%)
	PV	41 (20.9%)
	IgA pemphigus	1 (0.5%)
Subepidermal group (n=129)	BP	114 (58.1%)
	CBDC	1 (0.5%)
	EBA	5 (2.5%)
	DH	2 (1%)
	LPP	2 (1%)
	LIGA	3 (1.5%)
	Paraneoplastic pemphigus	1 (0.5%)
	Senear Usher syndrome	1 (0.5%)

Since BP, PV, and PF were clearly the most predominant (177 out of 230) diseases, we further analyzed their distribution across various age groups and genders.

The demographic features of the pemphigus and BP group of patients are summarized in Table 2.

**Table 2:** The demographic features of pemphigus and BP group of patients are summarized in this table PV: Pemphigus vulgaris; BP: Bullous pemphigoid; PF: Pemphigus foliaceus; IQR: Interquartile range

Variables		PV	BP	PF
Age in years (Mean±SD)		44±12	65±15	56±15
Gender	Male (n%)	15 (37%)	57 (50%)	5 (23%)
	Female (n%)	26 (63%)	57 (50%)	17 (77. %)
Duration in months (Median, IQR)		3,6	4,4	4,6

Among the 101 patients in the geriatric age group (aged 60 years and above), the most common autoimmune bullous disease was BP, affecting 82 patients (81.1%), followed by PV in 20 patients (19.8%) and PF in 11 patients (10.8%). In the adult age group, BP was also the most prevalent condition, diagnosed in 112 patients (63.27%), followed by PV in 41 patients (23.16%) and PF in 22 patients (12.4%). Among pediatric patients, only two cases were recorded, both of which were BP. Regarding gender distribution, PV and PF showed a predominance among females, whereas BP was evenly distributed between males and females.

McNemar's test was employed to assess the concordance in diagnostic accuracy between the assorted combinations of clinical diagnosis, HP, and DIF. This test is designed specifically for paired and nominal data; it is appropriate to use this test in assessing the variances in the responses when two related diagnoses or diagnostic assessments are being compared. Table 3 shows the concordance and discordance ratios among various diagnostic modalities.

**Table 3:** This table shows the concordance and discordance ratios among various diagnostic modalities DIF: Direct immunofluorescence; HP: Histopathology

Diagnosis Modality	Number (Percent) Matching the Final Diagnosis	Concordance Ratio	Discordance
Clinical diagnosis/Final diagnosis	133 (67.8%)	0.68	0.32
Histopathological diagnosis/Final diagnosis	176 (89.7%)	0.90	0.10
DIF diagnosis/Final diagnosis	170 (86.7%)	0.87	0.13
Clinical/HP	129 (65.82%)	0.66	0.34
HP/DIF	155 (79.08%)	0.79	0.21
DIF/Clinical	123 (62.46%)	0.62	0.38
Clinical & HP/Final diagnosis	183 (93.37%)	0.93	0.07
Clinical & DIF/Final diagnosis	181 (92.35%)	0.92	0.08
Clinical & HP /Clinical & DIF	180 (92.35%)	0.92	0.08
Clinical+HP+DIF/Final diagnosis	192 (97.9%)	0.98	0.02

Table 4 shows the statistical comparison of diagnostic concordance between different combinations of clinical, histopathological, and immunofluorescence modalities.

**Table 4:** Statistical comparison of diagnostic concordance between different combinations of clinical, histopathological, and immunofluorescence modalities Comparison of diagnostic modality combinations using McNemar’s test to assess statistical significance in concordance with the final diagnosis. A p-value <0.05 indicates a significant difference in diagnostic accuracy between the compared methods. The “Better method” column identifies the approach with higher concordance when a statistically significant difference was observed. DIF: Direct immunofluorescence; HP: Histopathology

Combination	P value	Interpretation	Better method
Clinical vs histopathological	< 0.0001	Significant	Histopathological
Clinical vs DIF	< 0.0001	Significant	DIF
Histopathological vs DIF	0.3173	Not significant	None
Clinical vs clinical & histopathological	0.0143	Significant	Clinical & histopathological
Clinical vs clinical & DIF	0.0016	Significant	Clinical & DIF
Histopathological vs clinical & DIF	< 0.0001	Significant	Clinical & DIF
Clinical & histopathological vs clinical & DIF	0.2850	Not significant	None
Clinical vs clinical + HP + DIF	< 0.0001	Significant	Clinical + HP + DIF
Histopathological vs clinical + HP + DIF	< 0.0001	Significant	Clinical + HP + DIF
DIF vs clinical + HP + DIF	< 0.0001	Significant	Clinical + HP + DIF

**Figure 1:**
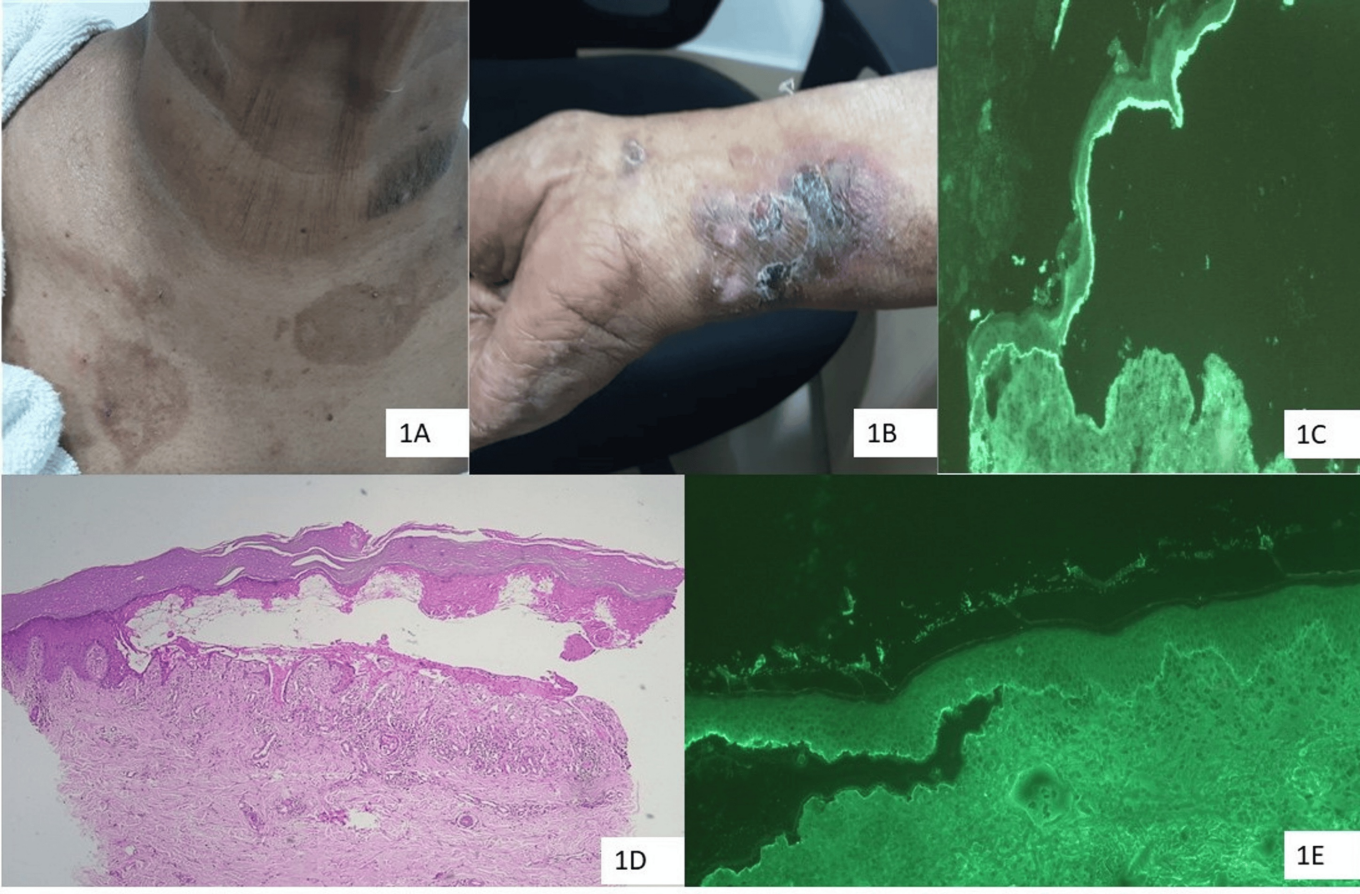
The clinical, histopathological and DIF images of an elderly patient suffering from BP A, B: Images of a 76-year-old female who suffered from a cerebrovascular accident nine months ago and then started developing tense, fluid-filled blisters for three months on a pruritic eczematous base C, E: DIF images of the patients demonstrating IgG and C3 deposition at the BMZ D: HPE (H&E stain on 40X) image showing subepidermal blister in the tissue sections of the same patient DIF: Direct immunofluorescence; BP: Bullous pemphigoid; IgG: Immunoglobulin G; BMZ: Basement membrane zone; HPE: Histopathological examination; H&E: Hematoxylin and eosin

Figure 1 (A, B, C, D, and E) shows the clinical, histopathological, and DIF images of an elderly lady with BP.

In four patients, the diagnosis remained uncertain despite clinical evaluation, HP, and DIF. It was ultimately confirmed through salt-split DIF, and all these patients were diagnosed with epidermolysis bullosa acquisita (EBA).

## Discussion

The objective of this study was to assess the diagnostic agreement between clinical examination, histopathological findings, and DIF in identifying ABD at a tertiary dermatological center in Kolkata, India.

The demographic analysis of our cohort revealed that BP was prevalent in both the geriatric and adult age groups. For the geriatric population, these findings align with existing literature [4]. However, among adults, previous studies have commonly reported PV as the most frequent diagnosis [5]. Our findings differ, which may be attributed to the rising overall prevalence of BP [6]. Regarding gender distribution, our study observed a higher prevalence of PF and PV in females, while BP affected males and females equally. This female predominance aligns with findings from other studies, suggesting potential gender-related susceptibility factors in ABD [7].

One of the major strengths of this study lies in the remarkable diagnostic agreement achieved by combining clinical evaluation with histopathological and DIF results. Clinical and histopathological assessment together showed a 90% concordance with the final diagnosis, while clinical evaluation paired with DIF reached 85%. Importantly, when all three diagnostic tools-clinical assessment, HP, and DIF were used in tandem, the concordance rose to an impressive 98%. This underscores the critical value of an integrated diagnostic approach for ABD. The high level of agreement not only illustrates how HP and DIF complement clinical findings but also significantly improves diagnostic precision. Previous studies have similarly demonstrated that while clinical evaluation is foundational, histopathological and immunofluorescence analyses are indispensable for definitive diagnosis and classification of ABD [8].

DIF emerged as an essential tool in distinguishing between intraepidermal and subepidermal bullous disorders. The distinct immunoglobulin deposition patterns, intercellular immunoglobulin G (IgG) and C3 in pemphigus variants, and linear IgG and C3 along the basement membrane in BP enabled accurate classification. Additionally, the presence of other immunoglobulins, such as IgM and IgA, in certain patients offered even greater diagnostic specificity, enhancing the ability to differentiate between these complex autoimmune conditions. These findings corroborate the established utility of DIF in ABD diagnosis, where it not only confirms the presence of autoantibodies but also aids in identifying specific disease subtypes [9].

In a similar retrospective study by Brar et al., the histopathological spectrum of these disorders was analyzed, and the diagnostic utility of DIF on both snap-frozen and paraffin-embedded tissue sections was assessed, particularly in differentiating autoimmune bullous disorders from connective tissue diseases of the skin [10]. A three-year prospective study (2017-2019) involving 27 biopsies was conducted, supplemented by a retrospective analysis of 25 biopsies collected between 2014 and 2017. All samples underwent HPE and DIF testing. PV emerged as the most frequently diagnosed condition in both phases, accounting for 37% and 36% of cases in the prospective and retrospective groups, respectively. DIF performed on snap-frozen sections demonstrated a higher specificity (81.25%) compared to paraffin-embedded sections, which showed a specificity of 66.6%. Additionally, the positivity rate for DIF was notably higher on snap-frozen sections (81.25%) than on paraffin-embedded ones (43.75%) in the prospective analysis. These findings highlight the superior diagnostic value of DIF when applied to snap-frozen tissue, especially in cases where clinical and histological features are inconclusive. A comprehensive diagnosis is most reliably achieved through the integration of clinical assessment, HP, and immunofluorescence findings [10].

In another retrospective cross-sectional study, conducted at the University of Medicine and Pharmacy in Ho Chi Minh City, Vietnam, the diagnostic concordance among these three modalities was assessed in 92 patients diagnosed with ABD between September 2019 and September 2021 [11]. Clinical histories, H&E-stained sections, and DIF results were reviewed by pathologists affiliated with the institution. Patients were categorized into intraepidermal and subepidermal blistering subgroups. When paired comparisons were made between diagnostic methods using McNemar’s test, no statistically significant differences were observed (p > 0.05). Overall, a moderate level of agreement was noted across all diagnostic approaches. Within the intraepidermal blister subgroup, HP and DIF demonstrated comparable diagnostic accuracy, both outperforming clinical diagnosis alone. In contrast, among the subepidermal blister subgroup, no significant differences in accuracy were identified between any diagnostic method pair. The highest concordance was observed in intraepidermal cases, while concordance was limited in subepidermal cases. These findings indicate that HP is particularly effective for diagnosing intraepidermal blistering diseases, whereas DIF remains essential for improving diagnostic precision, especially in subepidermal variants, within this Vietnamese patient population [11].

In a study analyzing 100 biopsy specimens over two years, the sensitivity and pattern of DIF were evaluated alongside clinical and histopathologic correlations [12]. Concordant DIF findings were observed in 89 cases, with sensitivity rates of 94.44% for pemphigus (51/58), 84% for BP (21/25), and 100% for both dermatitis herpetiformis (DH) (2/2) and linear IgA disease (LIGA) (1/1). DIF negativity was reported in 11 histologically confirmed cases, attributed to factors such as absence of epidermis (four cases), sampling or technical errors (three cases), and prior treatment (four cases). The findings underscored the diagnostic utility of DIF in immunobullous diseases, particularly where clinical and histopathologic features overlap, while highlighting sampling errors as a key cause of false-negative results [12].

In a retrospective analysis of 197 cases clinically diagnosed with vesiculobullous dermatitis, histopathological slides stained with H&E were re-evaluated, and all cases had undergone DIF examination [13]. Statistical analysis, including Kappa statistics, was performed using SPSS software to assess diagnostic concordance. Agreement between clinical and histopathological/DIF diagnoses varied widely, with a Kappa value of 0.29. Concordance was highest in PV (58.8%) and PF (53.8%), followed by BP (37.9%) and DH (5.2%). Among the less common conditions, concordance was 50% in linear IgA bullous dermatitis (two cases), 100% in Grover's disease (one case), 40% in EBA (five cases), and 0% in Hailey-Hailey disease (one case). The findings suggest reasonable diagnostic concordance in major pemphigus and pemphigoid variants, while highlighting poor agreement in DH. These results reinforce the necessity of incorporating DIF alongside light microscopy for definitive diagnosis, as histopathological findings alone may be insufficient [13].

A total of 215 skin biopsies from patients with suspected immune-mediated vesiculobullous diseases, vasculitis, or dermatoses were examined using HP and DIF for the presence of IgG, IgA, IgM, and complement component C3 deposits [14]. DIF yielded positive findings in 103 cases, with a high level of concordance observed between clinical, histological, and immunofluorescence results (observed agreement = 93.4%, Kappa = 0.90; 95% CI: 0.86-0.94). The overall sensitivity of DIF in immune-mediated skin conditions was 98.0%. Specifically, DIF positivity was found in 98.1% (52/53) of pemphigus cases and 96.0% (24/25) of BP cases. None of the clinically suspected DH cases showed positive DIF findings. A positive lupus band test was noted in all nine lupus erythematosus cases, and DIF positivity was observed in all ten cases of Henoch-Schönlein purpura. In 110 cases, negative DIF results were instrumental in excluding immune-mediated vesiculobullous diseases, lupus erythematosus, and vasculitis, with final diagnoses made based on clinical and histopathologic assessment. These findings underscore the utility of DIF as a valuable adjunct in the diagnosis and classification of autoimmune dermatological conditions, particularly when clinical or histological features are inconclusive, with optimal diagnostic accuracy achieved through an integrated approach [14].

The elevated prevalence of BP in our cohort, particularly among both adults and the elderly, emphasizes the critical importance of increased clinical vigilance and prompt diagnostic measures within this demographic. Early and precise diagnosis is crucial for initiating timely immunosuppressive treatments, which play a key role in reducing disease-associated morbidity and enhancing patients' quality of life. Furthermore, the observed gender predisposition suggests that clinicians should exercise greater suspicion for ABD, especially in female patients who present with blistering conditions.

This study does have some limitations. As a retrospective, single-center analysis based on convenience sampling, it is subject to selection bias and lacks a formal sample size calculation, which may affect the generalizability of the findings. The inability to apply standardized severity scoring systems, such as autoimmune bullous skin disorder intensity score (ABSIS) or pemphigus disease area index (PDAI), limited our ability to objectively correlate clinical severity with diagnostic modalities. Additionally, the use of existing medical records could have introduced selection and information biases, which might have impacted both the accuracy of clinical diagnoses and the comprehensiveness of the data. The exclusion of cases with inadequate biopsy samples may also underestimate the real-world diagnostic challenges encountered in routine practice. Moreover, excluding cases where prior treatments potentially altered DIF results may have skewed the outcomes by leaving out more severe or atypical presentations, thus limiting the scope of the findings. These limitations should be addressed in future prospective, multi-center studies with standardized protocols and complete data collection.

Future research should focus on conducting multi-center, prospective studies to confirm these findings in a wider range of populations and clinical environments. Further exploration into the molecular and genetic mechanisms of ABD, particularly within the Indian demographic, could reveal deeper insights into disease pathogenesis and uncover novel therapeutic targets. Additionally, assessing patient outcomes based on the diagnostic methods utilized would provide valuable information on how comprehensive diagnostic approaches influence treatment effectiveness and long-term prognosis.

## Conclusions

In conclusion, the study demonstrated that while histopathological evaluation provides a high level of diagnostic accuracy for intraepidermal autoimmune blistering disorders, its utility is more limited when applied to subepidermal variants. DIF was shown to be an indispensable adjunct in the diagnostic process, particularly for cases where clinical and histological findings are inconclusive or discordant. The integration of all three modalities, clinical examination, HP, and DIF, resulted in the most reliable diagnostic outcomes, underscoring the importance of a multimodal approach in the evaluation of ABD. These findings highlight the need for continued emphasis on comprehensive diagnostic protocols, especially in settings where diagnostic ambiguity can significantly impact treatment decisions and patient prognosis. Furthermore, the observed variation in concordance between subgroups suggests that disease subtype should guide the relative weighting of diagnostic tools in clinical practice.
